# Better understanding the provision and retention of podiatry specific diabetes education

**DOI:** 10.1186/1757-1146-8-S2-O44

**Published:** 2015-09-22

**Authors:** Julia Yuncken, Cylie Williams, Rene Stolwyk, Terry Haines

**Affiliations:** 1 Peninsula Health, Frankston, Victoria, 3199, Australia; 2 Faculty of Medicine, Nursing and Health Sciences, Monash University, Frankston, Victoria, 3199, Australia; 3 Allied Health Research Unit, Monash Health, Cheltenham, VIC, 3192, Australia

**Keywords:** Diabetes education, Podiatry education, education retention

## Background

Health literacy is fundamental to the provision of education within the health care setting. Poor health literacy regularly affects people's health and their ability to self care. Podiatrists regularly provide education to clients who have diabetes however little is known about the best method to do so, particularly when the client has complications or poor health literacy. This study aims to quantify what educational content is provided from podiatrists to clients with diabetes during assessments and routine appointments in order to further inform retention rates of education.

## Methods

This project was embedded within a prospective cohort study with two groups, three podiatrists and 24 clients. Participants were eligible to participate if they were a podiatrist at Peninsula Health or a client who attended podiatry consultations and had diabetes. Data collection included the Problem Areas in Diabetes Questionnaire (PAID), Montreal Cognitive Assessment (MoCA), information covered during the consultation and method of delivery and perceived key educational message from podiatrist and client perspectives.

## Results

The podiatrists ranged in experience from 1 to 11 years. There were 13 clients, mean age 59 (7.5) years, average duration of diabetes was 16 (11.5) years and MoCA score ≥26. The mean PAID measure was 25.58 (23.36), indicating participants were aware of their needs relating to diabetes and complications. During the consultations, the podiatrists on average covered 6 topics and in 100% of those consultations, this was delivered verbally. There were only four instances that the podiatrists and clients reported the same key educational message.

## Conclusions

This study identified that podiatrists cover a number of complex concepts during a diabetes related consultation. There was a disparity between key messages, meaning that what a podiatrist may think they have emphasised was not what the client heard or remembered when given verbal information only. This has potential negative implications for self-care and early identification of foot complications relating to diabetes. Podiatrists should consider how information is dissemination and provide written resources in plain English. Highlighting the key message may also assist the client at risk of future complications.

